# Research on Olympic medal prediction based on GA-BP and logistic regression model

**DOI:** 10.12688/f1000research.161865.2

**Published:** 2025-03-28

**Authors:** Sanglin Zhao, Jikang Cao, Jackon Steve

**Affiliations:** 1 School of Engineering Management, Hunan University of Finance and Economics, Changsha, Hunan, China; 2 School of Management, University of Khartoum, Khartoum, Khartoum, Sudan

**Keywords:** Genetic algorithm, Logistic regression, Virtual control group, Olympic medals, Coach effect

## Abstract

**Background:**

Predicting the number and distribution of Olympic medals in the future has become a hot topic, but predicting the number of Olympic medals is not easy and requires comprehensive consideration of multiple factors such as historical data, athlete performance, and host country effects.

**Method:**

This article uses the GA-BP algorithm model, combined with genetic algorithm (GA) and backpropagation neural network (BPNN), to optimize the weights and bias parameters of the BP neural network using the global search capability of genetic algorithm, thereby improving training efficiency and prediction performance. By estimating the number of Olympic gold medals and total medals, verifying the accuracy of the model, and predicting the medal table for the 2028 Los Angeles Olympics. Meanwhile, based on the synthetic control model, Estonia and China were selected as research subjects to construct a virtual control group and two experimental groups for analysis.

**Result:**

The experimental results showed that Estonia and China won more medals with a head coach than without one. In 1992, Estonia won 1 gold medal and 2 bronze medals under the guidance of excellent coaches, indicating the significant role of head coaches in improving athletes’ performance.

**Conclusion:**

This study provides valuable insights for the decision-making of the Olympic Committee, revealing key factors in medal distribution, optimizing the allocation of national strategic resources, and predicting the performance of countries at future Olympic Games.

## 1. Introduction

At the recent 2024 Summer Olympic Games in Paris, athletes from all over the world contributed countless wonderful moments to the audience. However, in addition to paying attention to the fierce competition of individual events, the audience also showed strong interest in the ranking of countries in the medal list. In this Olympic Games, the United States ranked first in the overall medal list with 126 medals, while China and the United States tied for the first place in the gold medal list, each winning 40 gold medals. France, the host country, ranked fifth in the gold medal list and fourth in the total medal list by virtue of its home court advantage. Although Britain ranked seventh in the gold medal list with only 14 gold medals, it rose to third place in the overall medal list with 22 silver medals and 29 bronze medals. In addition, small countries such as Albania, Cape Verde, Dominica and Saint Lucia won Olympic medals for the first time, among which Dominica and Saint Lucia won a gold medal respectively, and these achievements also triggered extensive discussions among the public and the media ( Olympics.com, 2024). ^1^

Medal list is not only a competition of sports strength between countries, but also reflects the resource input and strategic planning of countries in sports development. Continued attention to the medal list has aroused people’s interest in medal trend prediction, especially when the next Olympic Games is coming, and when the participation plans and athlete lists of various countries are gradually clear, predicting the medal distribution in the future Olympic Games has become a compelling topic. It is not easy to predict the number of Olympic medals, and many factors such as historical data, athletes’ performance and host country effect need to be considered comprehensively from multiple dimensions. Research shows that the host country can often significantly improve the medal performance, and this “home advantage effect” has been verified in the past summer and winter Olympic Games (Bernard & Busse, 2004). ^2^ In addition, the economic level and population size of a country are also significantly related to medal performance, for example, Johnson and Ali (2004). ^3^ Our research shows that there is a positive correlation between GDP and the number of medals.

Analyzing the data of previous Olympic Games can not only help us better understand the distribution law of medals, but also provide theoretical support for future medal prediction. This prediction model is not only of great significance for the media and the public to better understand the results of the Olympic Games, but also provides a reference for the strategic planning of Olympic committees in various countries, helping them to optimize the allocation of resources and concentrate on developing advantageous projects in future events.

In addition, with the continuous increase and adjustment of Olympic events, as well as the change of resources input in sports events in various countries, the medal distribution trend has become more complex and dynamic. Studies have shown that different events have different effects on the distribution of national medals. For example, swimming and track and field have made great contributions to the United States, while gymnastics and weightlifting have made significant contributions to China’s medals (Bian, 2015). ^4^ We need to explore the driving factors behind the medal performance of various countries, including the long-term investment of the country in sports development, the individual performance of athletes, the ability of the coaching team, and the influence of emerging projects on the medal list. This can not only help to predict the medal distribution, but also reveal the competitive advantages of different countries in specific events, and provide more comprehensive guidance for future Olympic preparations.

Through these data-driven analysis, medal prediction is no longer just a simple estimation of the results, but an important tool to provide insight and decision-making basis for the national sports strategy.

In recent years, the combination of genetic algorithm (GA) and backpropagation neural network (BPNN) has attracted much attention in the field of prediction. As an optimization algorithm, GA effectively solves the problems of BPNN being prone to local optima and slow convergence, while BPNN performs well in multiple prediction tasks with its powerful nonlinear fitting ability. ^10– 12^ The GA-BP model is widely used in engineering performance and parameter optimization, material performance and process optimization, environmental and safety monitoring, energy and power systems, as well as intelligent control and automation. ^13– 15^

In the engineering field, the GA-BP model has been used to predict the performance and emissions of heavy-duty hydrogen internal combustion engines, detect bolt loosening faults, predict SRC shear beam strength and classification failure modes, etc. Dezhong Ning et al.’s ^11^ research has demonstrated its application in optimizing the performance of hydrogen internal combustion engines. In materials science, this model successfully predicted the wear of ball bearings, the forming quality of aluminum alloy additive manufacturing, and the strength of microwave sintered ceramics, improving the accuracy of process control and material performance prediction. ^16– 18^

In terms of environment and safety, the GA-BP model constructs a gas sensor pressure compensation model, which improves the accuracy of gas leak detection; It also accurately predicted the heat release rate and temperature field of subway tunnel fires, providing support for safe tunnel operation. In addition, it is also used to predict harmful gas emissions in marine areas and the failure pressure of urban water supply pipelines, contributing to environmental protection and public safety.

In the field of energy and power systems, the application of GA-BP models is increasing, such as the distributed photovoltaic maximum power point prediction method, which improves the efficiency of photovoltaic power generation; **Ziqiang Lu** et al.’s ^12^ research demonstrated its excellent performance in detecting bolt loosening faults in power grid transmission towers. This model has also been used for battery state estimation, smart grid load forecasting, etc., promoting the intelligent development of energy systems.

In the field of intelligent control and automation, GA-BP models also play an important role, such as the intelligent agricultural irrigation warning system, which achieves accurate prediction and intelligent control of irrigation flow; **Qiang Guo** et al. ^10^ optimized the machine tool screw conveyor to improve conveying efficiency and reduce energy consumption. In addition, the model has also been used for building energy-saving renovation optimization and intelligent manufacturing process control, promoting the advancement of intelligent control and automation technology.

In summary, the combination of GA and BP models has shown broad application prospects and significant advantages in the field of prediction, improving the convergence speed and prediction accuracy of the model, and effectively avoiding local optimal solution problems. In the future, with the improvement of algorithms and the enhancement of computing power, the application of GA-BP models in the field of prediction will be more extensive and in-depth, providing strong support for the intelligent development of various fields.

The application of GA-BP model in Olympic medal prediction can fully utilize diverse data such as historical medal data, athlete information, and project advantages. By optimizing the initial weights and biases of BP neural network through GA, it avoids BPNN falling into local optima during training, thereby improving the convergence speed and prediction accuracy of the model. In addition, the GA-BP model can flexibly handle the differences between different sports projects, refine modeling based on the characteristics of different projects, and further improve the accuracy and reliability of predictions.

Therefore, in combination with the actual demand for Olympic medal prediction, using the GA-BP model for prediction can not only comprehensively consider various influencing factors, but also more accurately capture the complex relationships between data, providing a more scientific and effective tool and method for Olympic medal prediction.

## 2. Data selection and analysis

### 2.1 Data preprocessing

**Figure 1.**
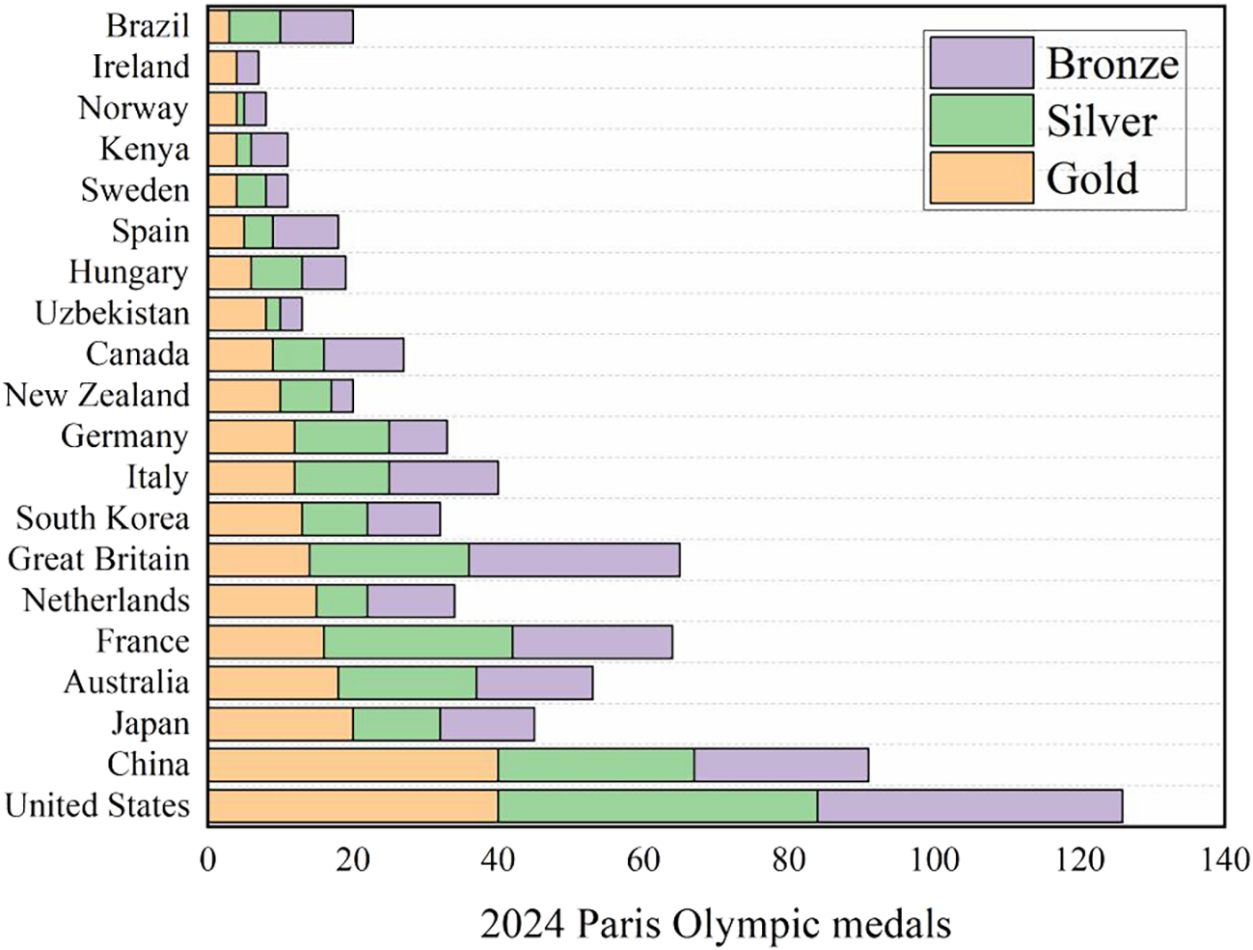
Medals for the 2024 Paris Olympic Games.

The data of multiple data sets (data set references) are merged into an excel form, and descriptive statistical analysis is made. Taking the 2024 Paris Olympic Games as an example, as shown in Figure 1, and the missing values are replaced and filled. The descriptive statistical results are shown in Tables 1- 2 and Table 3 model accuracy evaluation in the Table 4.

**Table 1.** Symbol description table.

Symbol	Meaning	Unit
N	Number of countries	/
$M_{i}$	Total number of medals in country I	trunk/horsewhip/used in connection with coins
$G_{i}$	Total number of gold medals in country I	trunk/horsewhip/used in connection with coins
$S_{i}$	Total number of silver medals in country I	trunk/horsewhip/used in connection with coins
$B_{i}$	Total number of bronze medals in country I	trunk/horsewhip/used in connection with coins
${\text{GOLD}}_{i}$	Gold medal changes in country I	trunk/horsewhip/used in connection with coins
HOST	Is it the host country?	{0,1}
Total events	Total projects	individual
NOC	Country name	N/A
X	sample	N/A
Year	age	N/A
Gold_Increment	Gold medal increment	trunk/horsewhip/used in connection with coins

**Table 2.** Descriptive statistical results.

Descriptive statistics											
	N	minimum value	maximum	total	average value	standard deviation	variance	skewness		kurtosis	
Year	6300	1896	2024	12364800	1962.67	38.762	1502.461	-.151	.031	-1.201	.062
Gold	6300	0	83	5809	.92	4.348	18.909	8.887	.031	102.564	.062
Silver	6300	0	seventy-eight	5777	.92	3.786	14.330	8.117	.031	93.295	.062
host	6300	0	one	24	.00	.062	.004	16.113	.031	257.709	.062
Total sports	6300	11	33	132510	21.03	5.339	28.503	.375	.031	-.463	.062
Bronze	6300	0	77	6292	1.00	3.739	13.978	6.849	.031	67.209	.062
Total disciplines	6300	10	50	181860	28.87	9.437	89.063	.535	.031	-.391	.062
Total events	6300	43	339	1210860	192.20	82.039	6730.428	.293	.031	-1.141	.062
Total	6300	0	231	17878	2.84	11.548	133.349	7.925	.031	85.435	.062
Gold_Change	6300	-80	83	0	.00	3.815	14.554	-.348	.031	147.811	.062
Gold_Increment	6300	-80	83	0	.00	3.815	14.554	-.348	.031	147.811	.062

**Table 3.** Classification and summary of the average medals in Olympic events.

Sport	Medals	Total
Archery	17.7	81.75
Art Competitions	6.556	159.944
Artistic Gymnastics	5.053	78.158
Athletics	134.35	1040.15
Basketball	43.278	158.056
Beach Volleyball	4.706	28.824
Boxing	25.1	122.65
Canoe Slalom	1.471	8.647
Canoe Sprint	5.25	23
Canoeing	34.65	180.3
Cycling	51.4	315.05
Diving	20.35	100.2
Equestrian	4.35	29.3
Equestrianism	38.5	224.65
Fencing	73.35	375.95
Figure Skating	2.857	six
Football	61.85	204.35
Golf	2.947	17.158
… …	… …	… …

**Table 4.** Model evaluation index parameters.

Variable	Definition	Computing formula
MSE	Mean-squared error	$\frac{\sum_{i=1}^{n}{\left(y_{i}-\bar{y_{i}}\right)}^{2}}{n}$
R2	Goodness of fit	$1-\frac{\sum_{i}{\left({\hat{y}}_{i}-y_{i}\right)}^{2}}{\sum_{i}{\left(y_{i}-\bar{y}\right)}^{2}}$
Evar	Explanatory variance value	$1-\frac{\sum_{i=1}^{n}{\left(\left(y_{i}-\hat{y}\right)-E\left({\vec{y}}_{-}\hat{y}\right)\right)}^{2}}{\sum_{i=1}^{n}{\left(y_{i}-\bar{y}\right)}^{2}}$
MAE	Average absolute error	$\frac{1}{m}\sum_{i=1}^{m}|h\left(x_{i}\right)-y_{i}|$

### 2.2 Correlation analysis

The variables were analyzed for correlation, and Pearson correlation test was used because the data passed the normality test.

**Figure 2.**
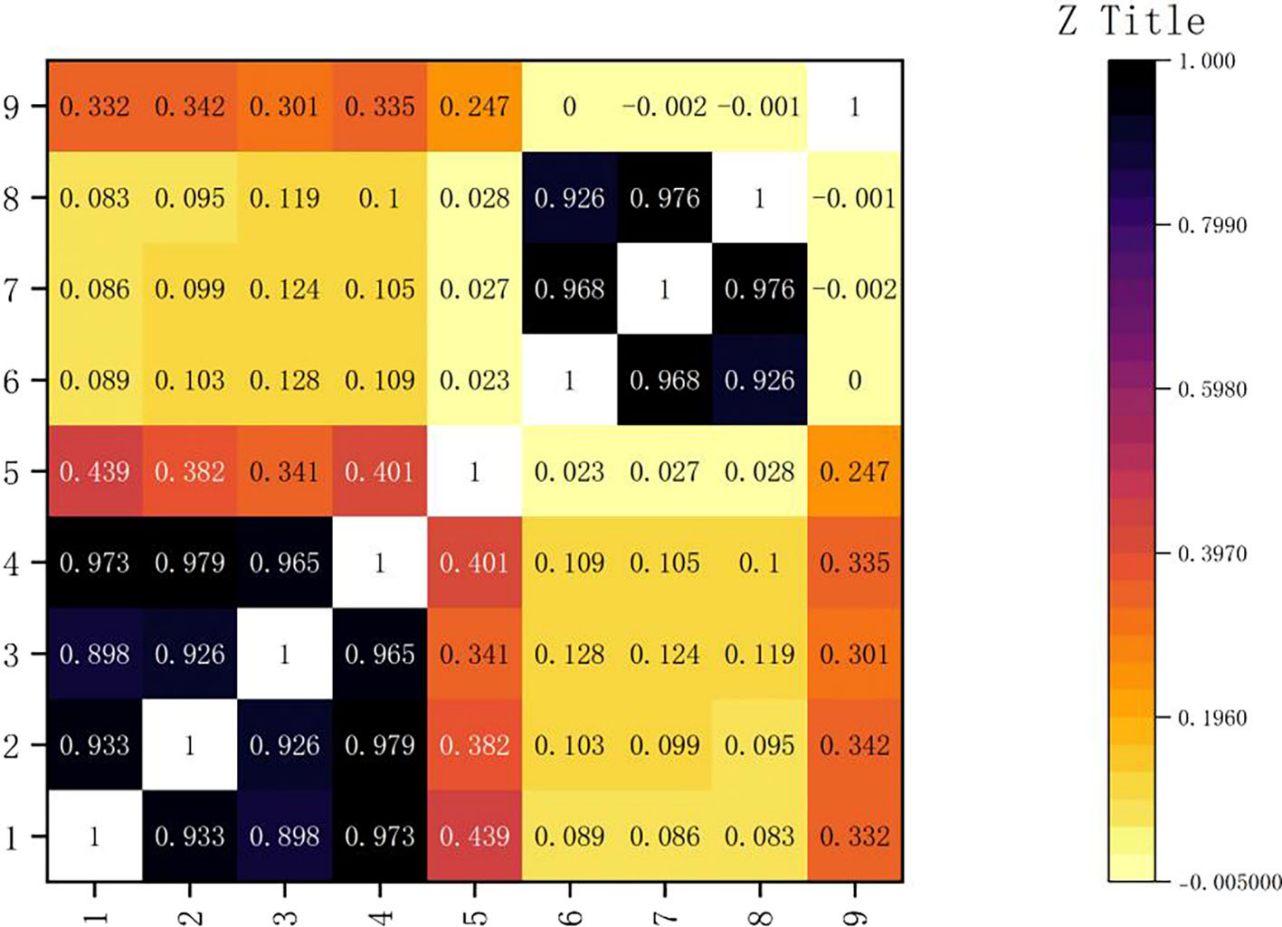
Correlation coefficient thermogram.

**Figure 3.**
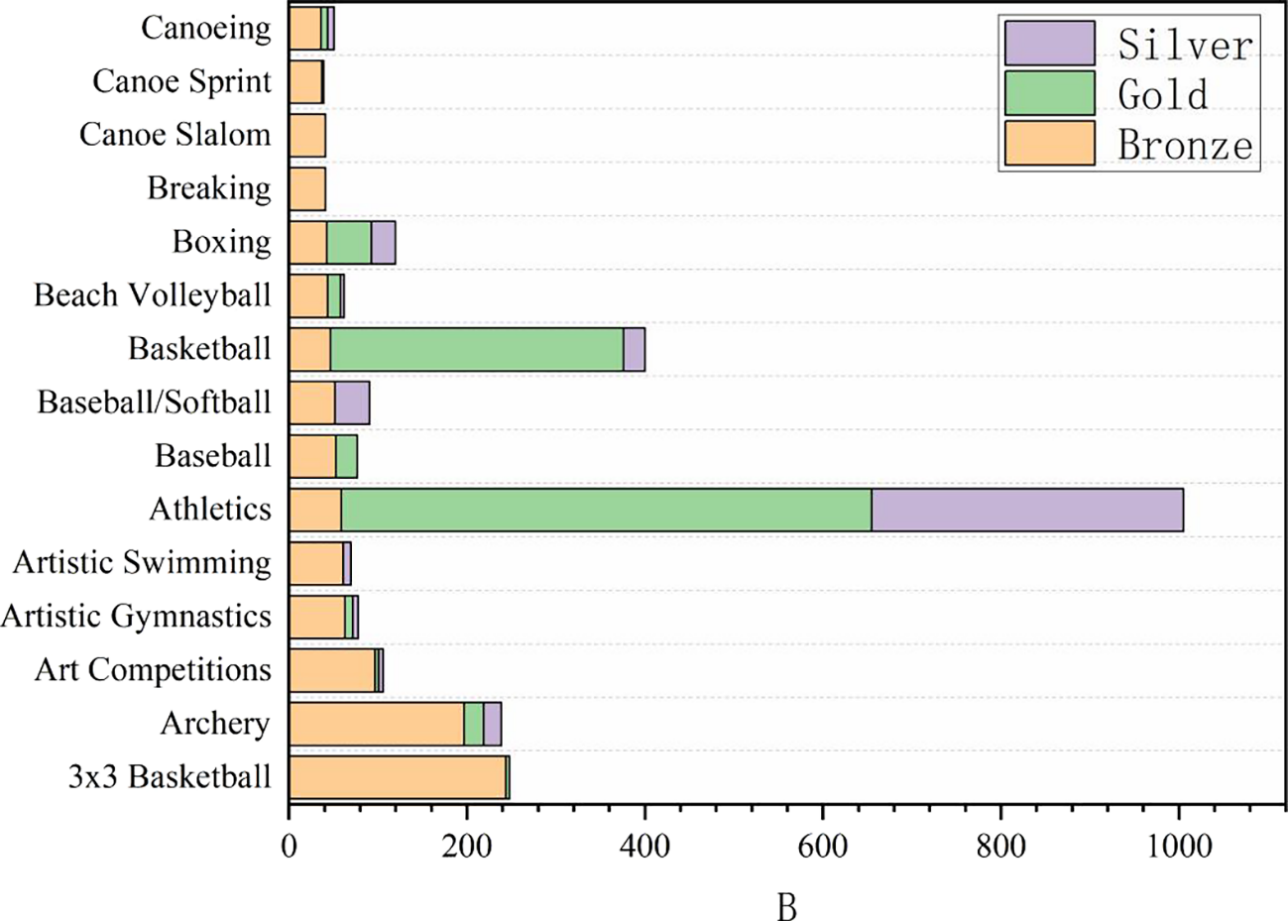
Proportion of awards in Olympic events.

The result is shown in the following Figure 2:

As shown in Figure 3, the axis of the icon lists the correlation coefficients of gold, silver, bronze and total medals. These correlation coefficients reflect the degree of correlation between the number of medals and other columns in the table. Among them, it is shown that there is a very close relationship between them. The correlation between the HOST variable and the total number of medals is 0.3, which shows that the athletes in the host country have the advantage of gold medal, but it is not significant. Through further analysis, we can find that gold medals occupy an important position in the total number of medals. In the Olympic Games, the gold medal is the symbol of the highest honor, and every participating country or region will strive for more gold medals. At the same time, although the number of silver medals and bronze medals is also included in the total number of medals, their value is relatively low. Therefore, the change of the number of gold medals will often affect the total number of medals. It can also be seen from the figure that the correlation between gold medals and silver medals and bronze medals is also high, which is 0.933 and 0.898 respectively, but it is still not as large as the correlation coefficient between gold medals and total medals, which further proves the important position of gold medals in the total number of medals. This also shows that in the Olympic Games, when countries compete for medals, they often pay attention to the number of gold medals, silver medals and bronze medals at the same time, but the gold medal is always the most important goal.

### 2.3 Analysis of the number of medals won in sports events

In the provided grouping summary table, the data show Medals and Total of different Sport. Through the analysis of these data, we can draw the following rigorous, refined, accurate, complete, coherent and logical interpretations: from traditional Athletics and swimming to Cricket and Croquet. Medals, as a key index to measure the performance of each event, reveals the competitive situation and strength distribution among events. Total may reflect the participation, influence or overall scale of the events. In order to deeply analyze the competitive level and popularity of each event, swimming (83) and track and field (52, on average) are the two events with the largest number of medals, indicating that these two events have a very high level of competition and extensive participation. At the same time, basketball (11.5), boxing (18.5), gymnastics (if rhythmic gymnastics and competitive gymnastics medals are considered together, the total number is 50.5, but the analysis here is based on separate data) and other projects have also won more medals, revealing the popularity and competitiveness of these projects in the global scope.

### 2.4 Evaluation of indicators

## 3. Establishment and solution of medal prediction model

### 3.1 Establishment of medal prediction model

3.1.1 BP neural network model

BP network is a nonlinear system, and its most remarkable feature is its high adaptability (Sanglin Zhao, 2024). ^5^ The algorithm takes 100 samples of the research object as the input nodes of the neural network, and the expected results as the corresponding output nodes, calculates the weights and thresholds, and calculates the error between the actual results and the predicted results, as shown in Formula (1). Fitness function is the standard to measure whether the error value meets the requirements. For the calculation results that do not meet the requirements, the network will carry out error back propagation. Equation (2) is the correction of the hidden layer and the output layer. Through repeated iterations, if the error meets the expectations, the model is successfully established. $\mathrm{nX}=\left(x_{1},x_{2},\cdots ,x_{j},\cdots ,x_{n}\right)Y=\left(y_{1},y_{2},\cdots ,y_{j},\cdots ,y_{n}\right)$

$$E\left(\omega \right)=\frac{1}{2}\sum_{k,j}\left[y_{i}\left(n\right)-y_{i}^{\prime }\left(n\right)\right]$$ (1)

$$\Delta W_{i\bar{y}}=-\eta \frac{\partial E}{\partial W_{\mathrm{kj}}}$$ (2)

Where: the expected result is the prediction result, the learning rate is the hidden layer, the output value is the output layer, and the output value of this node is the correction. $y_{i}\left(n\right);\;y_{i}^{\prime }\left(n\right);\;\eta ;E;\;\Delta W_{\mathrm{kj}};\;\Delta W_{\mathrm{kj}}$ .

3.1.2 Genetic algorithm

**Figure 4.**
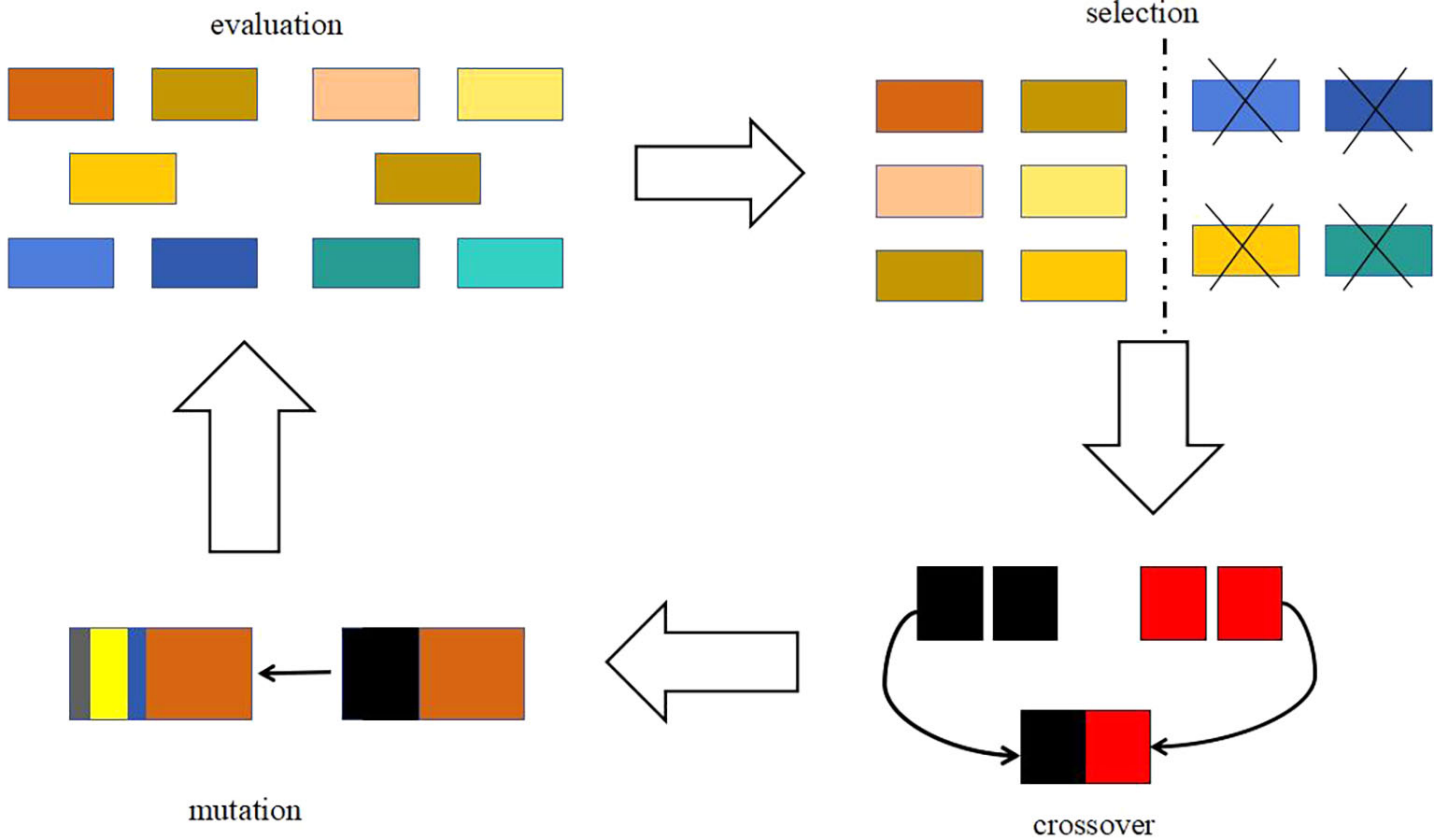
Schematic diagram of genetic algorithm.

**Figure 5.**
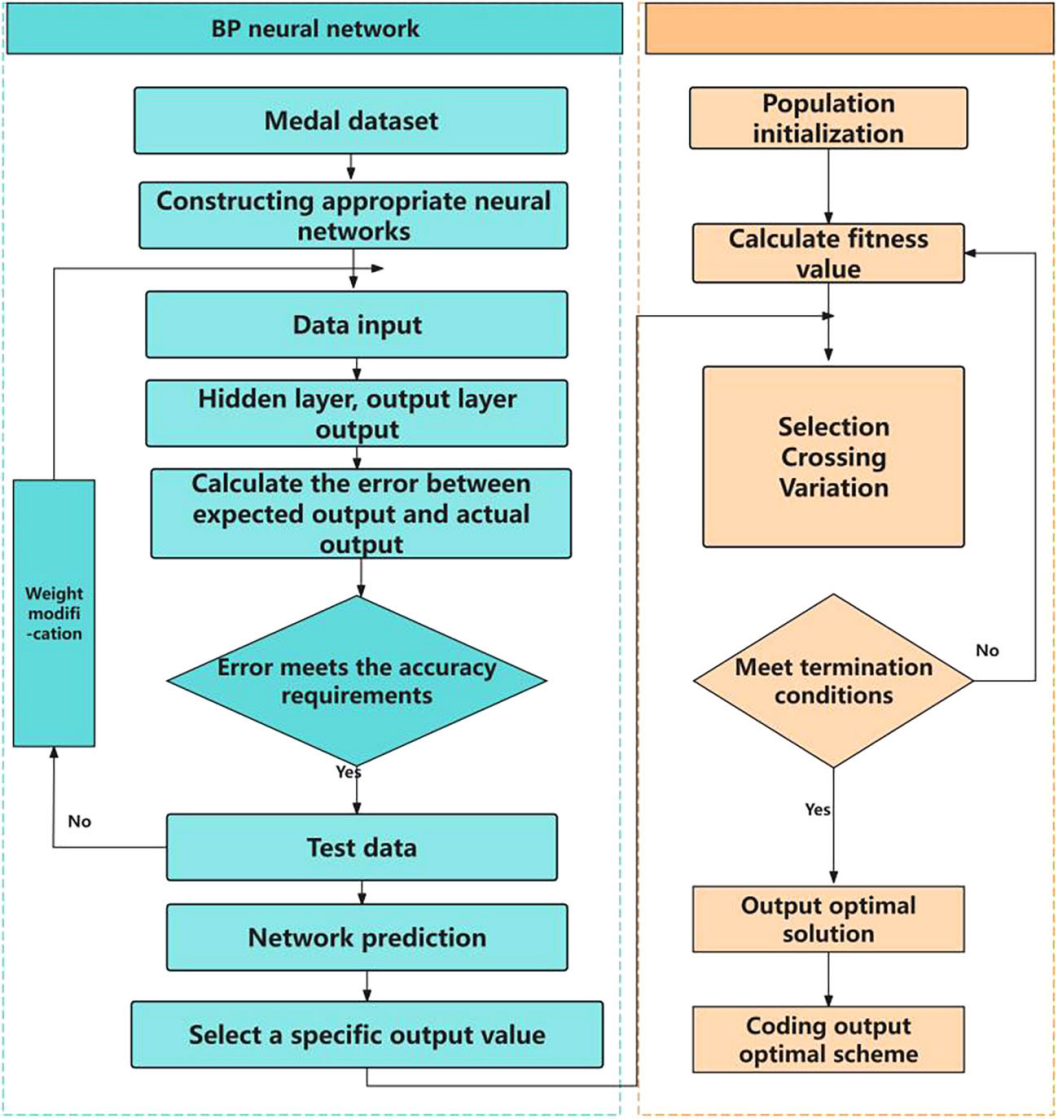
GA-BP flow chart.

The basic idea of genetic algorithm is a population that needs to be optimized. On the basis of the survival of the fittest, it evolves from generation to generation, leaving the evolved population with good fitness and inheriting it to the next generation. In this process, it needs to be crossed and mutated to produce a new population with better fitness. The optimization process is shown in Figures 4 and 5.

A. Chromosome coding

Floating-point coding is selected as the coding rule, which can obtain higher precision network weights and thresholds on the one hand, and a wider search range than before on the other hand, with the length s as shown in Formula (3). Where is the number of neurons in input layer, hidden layer and output layer.

$$s=n_{1}\times n_{2}+n_{2}\times n_{3}+n_{2}+n_{3}$$ (3)

B. Fitness function

During the training of a BP neural network, the network’s outputs and desired outputs are selected as training samples. After training, the resulting weights and thresholds are chosen as the optimal parameters. Genetic algorithm metrics are employed as indicators for the optimization process. Subsequently, the sum of squared errors between the predicted values and actual values is computed., as shown in the formula. $\bar{y}\left(k\right)y\left(k\right)$

$$J_{m}=\frac{1}{2}\sum_{k=1}^{N}{\left[\bar{y}\left(k\right)-y\left(k\right)\right]}^{2}$$ (4)

In this context:

*k* represents the logarithmic transformation of both input and output sampling data.

*n* denotes the total count of network output nodes.

The kth node’s anticipated output is compared against its predicted output value.

In order to avoid the phenomenon of dividing by zero, a value close to zero is introduced here, and the fitness function is Formula (5). $\bar{y}\left(k\right)y\left(k\right)\delta$

$$\mathrm{fit}={\left(J_{m}+\delta \right)}^{-1}$$ (5)

C. Choose

Let the population size be n and fi be the fitness, and this fitness is for the individual I in the population, then the probability of I being selected is shown in Formula (6).

$$P_{\mathrm{si}}=f_{i}/\sum_{i=1}^{n}f_{i}$$ (6)

D. Crossing

Arithmetic crossover method is widely used in individuals with floating-point crossover. Assuming that arithmetic crossover is performed between individuals, the new individuals generated after the operation are shown in the Formula (7). $x_{A}^{t}\;\text{and}\;x_{B}^{t}$

$$\left\{ \begin{array}{ll} x_{A}^{t+l}+\alpha x_{B}^{t}+\left(l-\alpha \right)x_{A}^{t} & \\ x_{B}^{t+l}+\alpha x_{A}^{t}+\left(l-\alpha \right)x_{B}^{t} & \end{array}\right.$$ (7)

Where: α is a random number evenly distributed in the interval [0,1].

E. Variation

In order to prevent premature phenomenon, based on a small range of random numbers, genetic variation occurs in a small range of probability, and the local search ability of the algorithm will also be strengthened in this step. At this time, it is assumed that the maximum and minimum values of the initial individuals of the population are respectively summed, and then the mutated gene is cut as shown in Formula (8). *x*_min_ and *x*_max_ then, the mutated gene can be seen in Formula (8)

$$x_{k}=x_{\min}+\beta \left(x_{\min}+x_{\max}\right)$$ (8)

Where: *β* is a random number evenly distributed in the interval [0,1]. Figure 4 is a schematic diagram of genetic algorithm, and Table 5 is a flow chart of combinatorial optimization model.

**Table 5.** Comparison chart of model accuracy.

Comparison of model accuracy	GA-BP	BP
R2	0.9881	0.9001
MSE	0.629	0.992
Evar	0.9975	0.9905
MAE	0.1338	0.3326

### 3.2 The solution of medal prediction model

**Figure 6.**
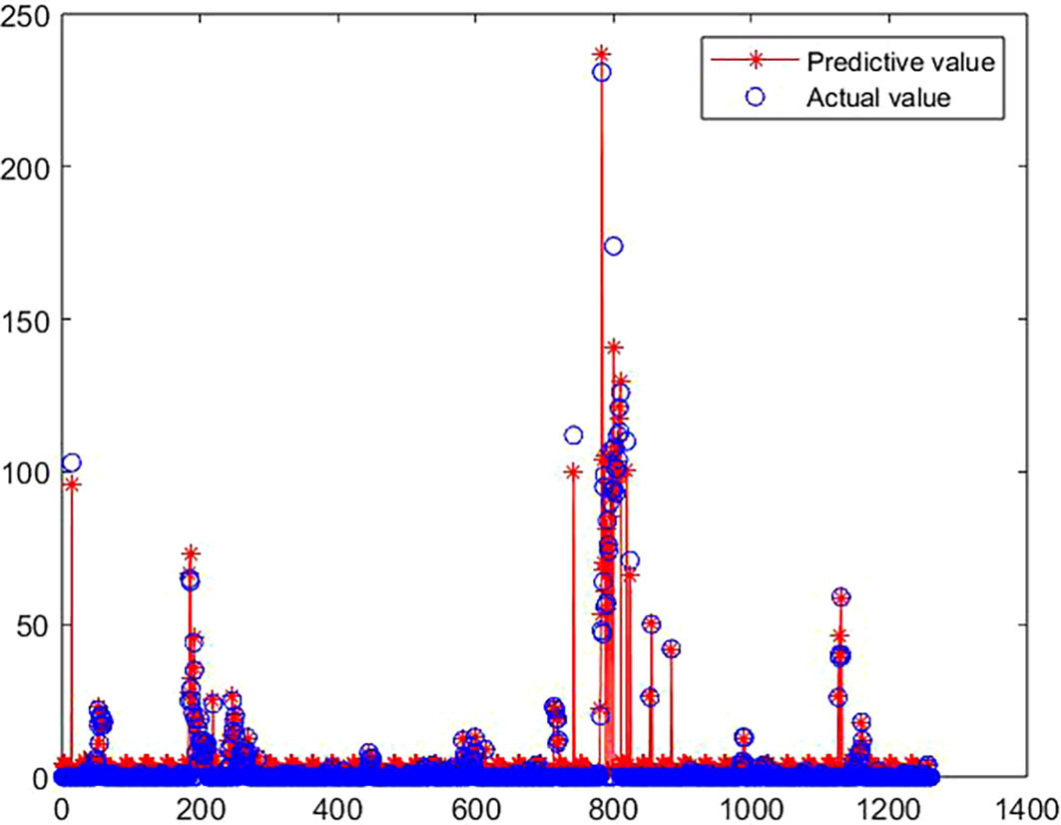
Comparison between predicted value and true value of GA-BP model.

**Figure 7.**
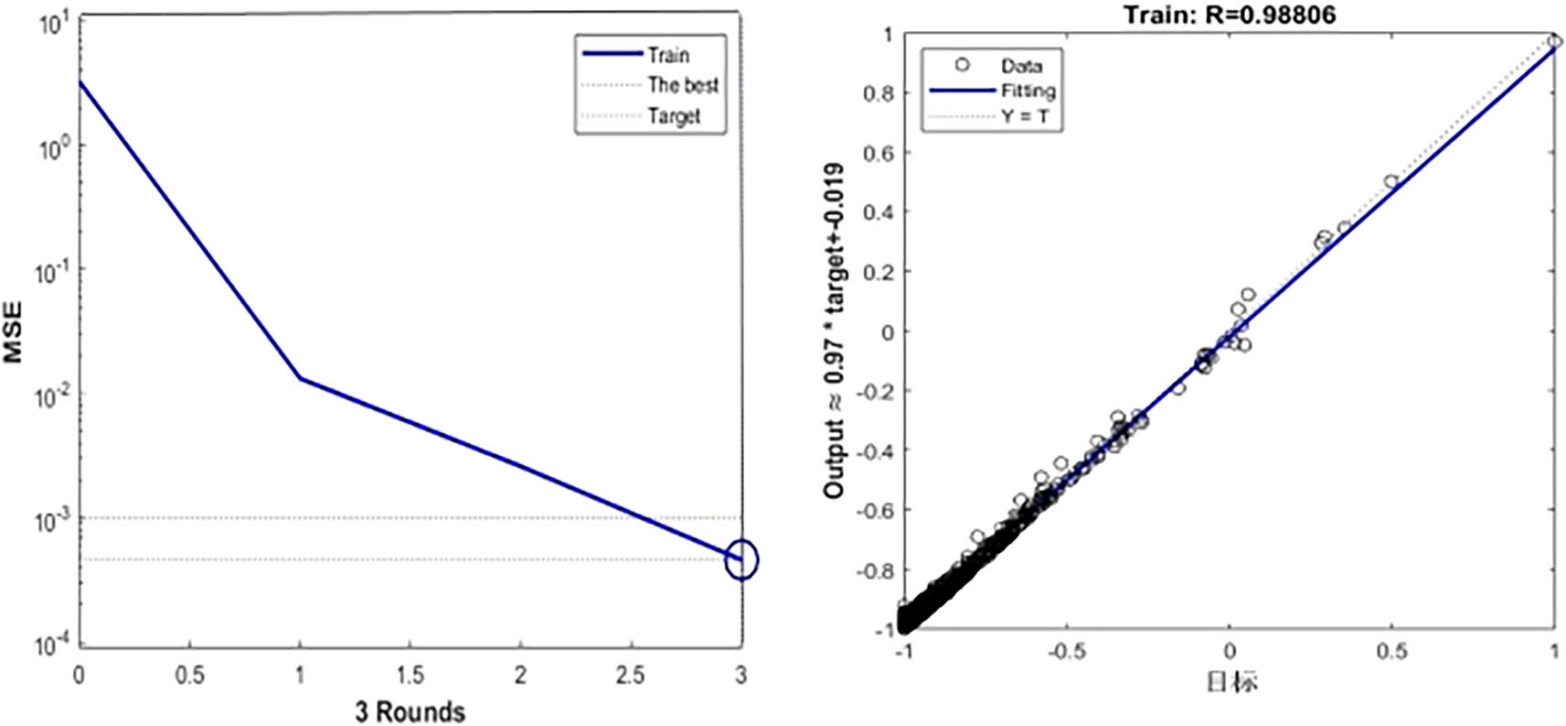
Model training process diagram.

Figure 6 shows the fitting effect of the prediction model:

Figure 6, Figure 7 and Table 5 show the training process of GA-BP algorithm model. According to the information analysis in the figure, the R value of BP algorithm model on the target data set is 0.9001, while the R value of GA-BP algorithm model on the target data set is 0.9881, which is closer to 1 than BP algorithm model, which indicates that the fitting degree of GA-BP model is higher than that of BP model. In addition, MSE can also be observed in Figure 7. The models of the two algorithms have different MSE values after different rounds of training. After three rounds of training, the MSE value of GA-BP algorithm reaches the minimum value of 0.629, which greatly reduces the mean square error compared with the BP algorithm model, which shows that the model is simpler and the prediction accuracy is higher. This paper shows the performance comparison results of GA-BP algorithm and BP algorithm on different data sets through several performance indicators. As can be seen from the figure, GA-BP algorithm is superior to BP algorithm in training error, R value, MSE value, Evar value and MAE value.

The prediction results are shown in Table 6:

**Table 6.** Forecast results of gold medals in various countries.

NOC	Gold_Predicted
United States	forty-two
China	37
South Korea	16
Australia	15
Japan	13
Germany	11
Netherlands	11
Great Britain	10
Italy	9
ROC	9
France	8
Brazil	7
New Zealand	7
Uzbekistan	7
Canada	6
Hungary	5
Kenya	5
Iran	4
Spain	4
Croatia	3
Georgia	3
… …	… …
Serbia	2

**Figure 8.**
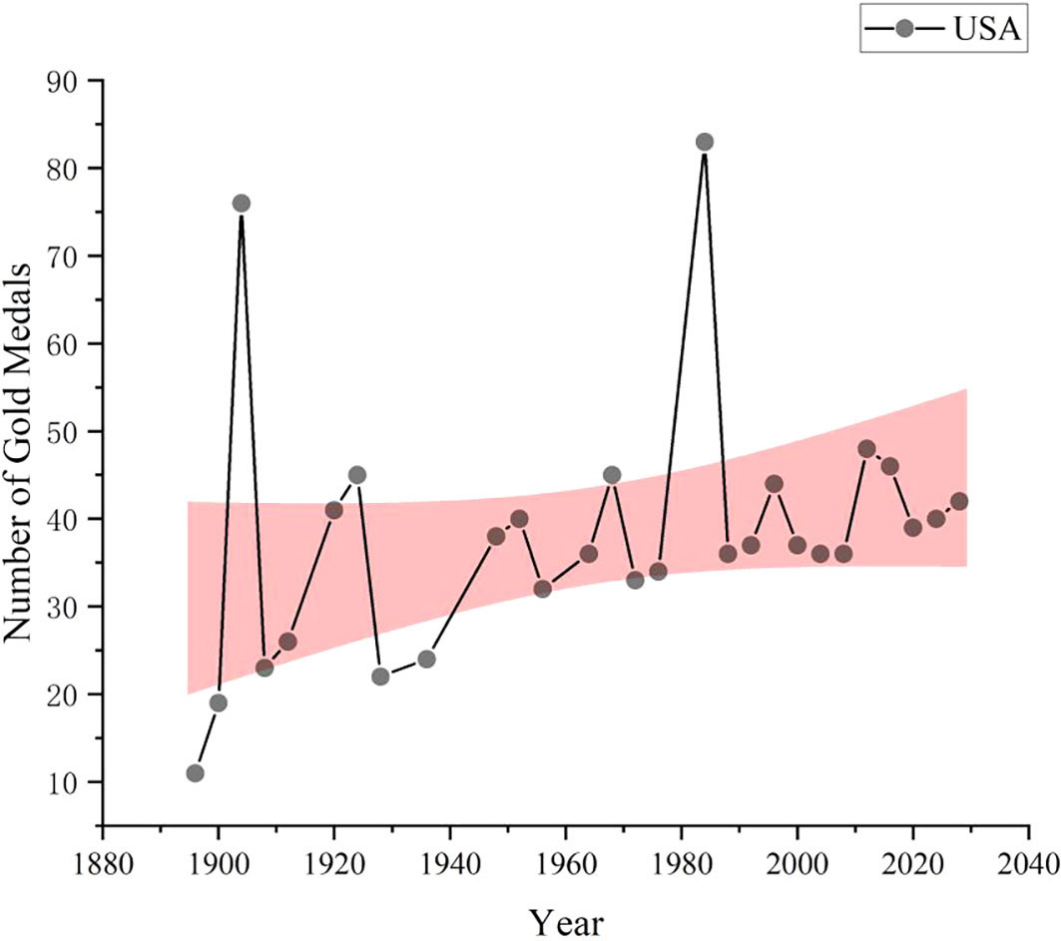
American gold medal forecast chart.

As shown in Figure 8, data collection and analysis based on GA-BP algorithm show that United States is expected to win 42 gold medals in the 2028 Los Angeles Olympic Games, ranking first in the gold medal list, followed by China with 37 gold medals and South Korea with 16 gold medals. The number of gold medals predicted by other countries is shown in the figure. As a global sports power, the United States has always performed well in international sports events such as the Olympic Games. The United States has strong competitiveness in various sports, especially track and field, swimming, gymnastics and other events, which are often the major events that produce gold medals. The number of gold medals predicted this time is as high as 42, which reflects the leading position of the United States in global sports competition. As a new sports power, China has performed better and better in international sports events such as the Olympic Games in recent years. The number of gold medals predicted this time is 37, although it is slightly less than United States, but it is still a very impressive number, which is far from the third place. China has traditional advantages in table tennis, badminton, diving, weightlifting, etc. At the same time, it is also striving to enhance the competitiveness of other new sports and strive to achieve the all-round development of national sports. Other countries such as Great Britain and Italy have relatively few gold medals. This does not mean that the sports strength of these countries is weak, but that the number of gold medals predicted may be low due to fierce competition or other factors. In addition, there are some countries behind, although the number of gold medals predicted is not much, but they may also make breakthroughs in some advantageous events.

### 3.3 Logistic Regression Prediction Model and Solution of First Gold Medal Countries

The sports events and winning trends are used as data training sets for logical probability regression, and the logical variables of winning gold medals or not are used as dependent variables for analysis.

$$P\left({\text{Medal}}_{i}=1\right)=\frac{1}{1+e^{-\left(\alpha +\beta_{1}\cdot {\text{Athletes}}_{i}+\beta_{2}\cdot {\text{Trend}}_{i}\right)}}$$ (9)

The binary logistic regression results in Tables 7, 8 and 9 show that:

**Table 7.** Likelihood chi-square ratio results.

Likelihood ratio chi-square value	P	AIC	BIC
115.633	0.000***	121.633	129.448

Note: * * *, * * and * represent the significance levels of 1%, 5% and 10% respectively.

The results of the model's likelihood ratio chi-square test show that the significance P value is 0.000***, which is significant horizontally and rejects the original hypothesis, so the model is effective.

**Table 8.** Model parameter settings.

Experimental group = 1.0	coefficient of regression	standard error	Wald	P	OR	95% confidence interval of OR value	
						upper limit	lower limit
constant	-0.561	0.485	1.342	0.247	0.57	0.221	1.475
Total	0.011	0.003	11.334	0.001***	1.011	1.005	1.017
Sport	-0.005	0.014	0.131	0.718	0.995	0.969	1.022

Dependent variable: y (whether winning or not)

Note: * * *, * * and * represent the significance levels of 1%, 5% and 10% respectively.

**Table 9.** Evaluation table of logistic regression model.

Accuracy rate	Recall rate	Accuracy rate	F1	AUC
0.72	0.72	0.75	0.715	0.802

The significance p value of Total is 0.001***, which is significant horizontally, rejecting the original hypothesis. Therefore, Total will have a significant impact on Y (whether it wins the prize or not), which means that the probability of Y (whether it wins the prize or not) is 1.099% higher than that of 0.0 for every additional unit of Total. Sport has no significant influence on Y (whether winning or not) (p=0.718).

ROC curve

**Figure 9.**
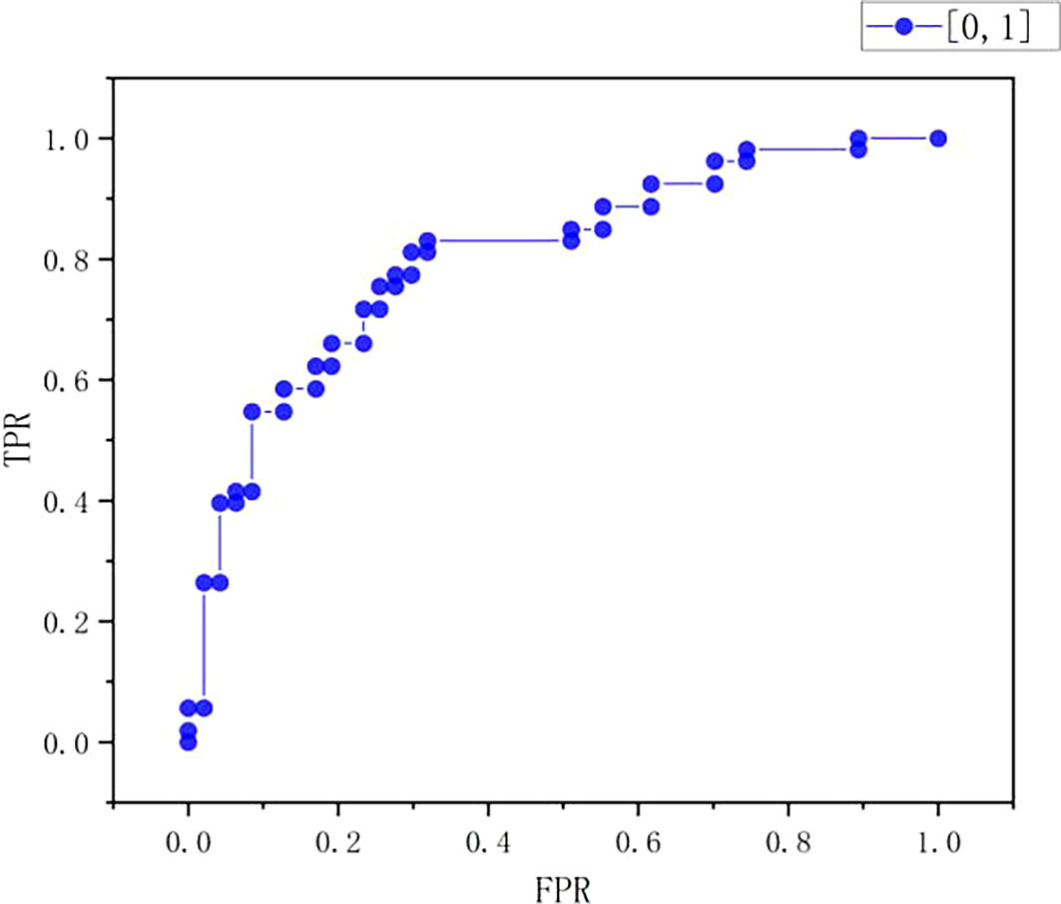
ROC curve.

**Figure 10.**
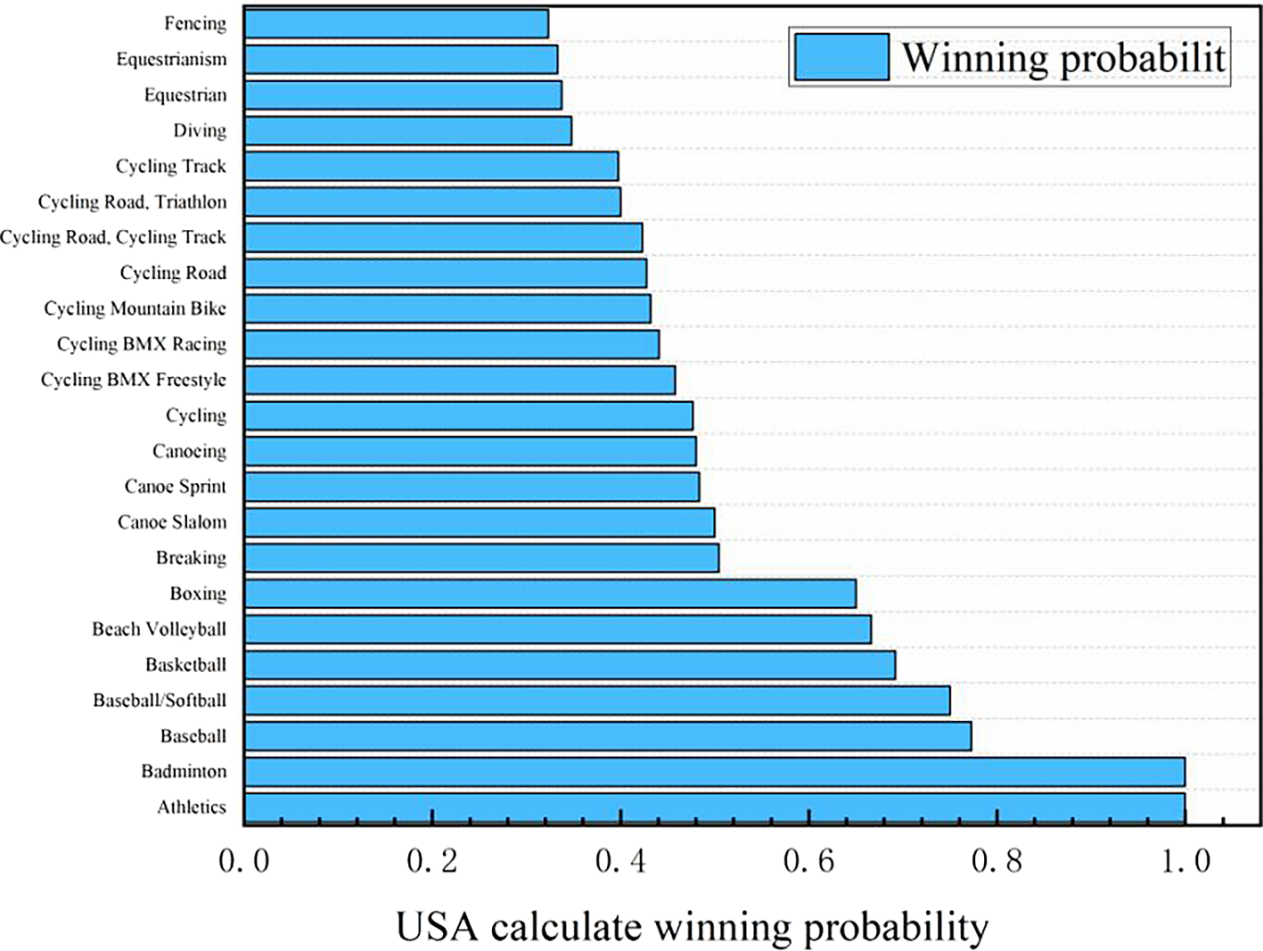
Predicting the probability of winning gold medals in American events (part).

**Figure 11.**
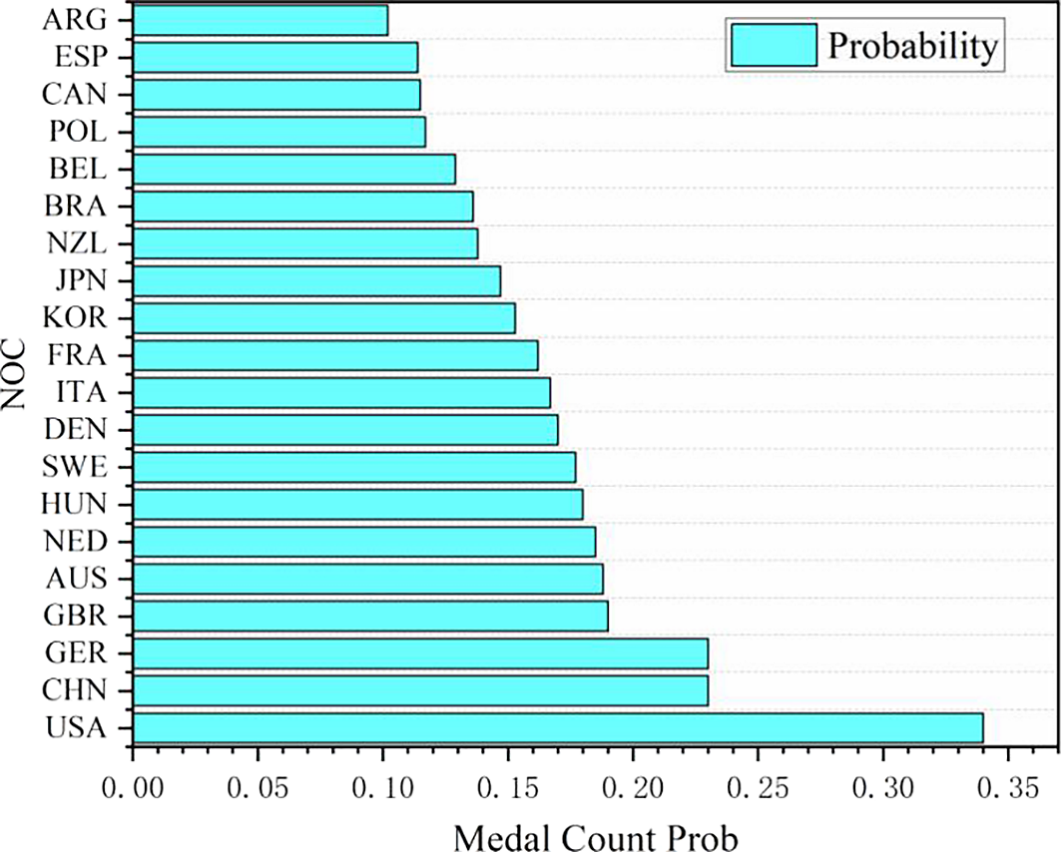
Probability map of winning gold by countries (some top countries).

As can be seen from Table 10 and Figures 9- 11, the true positive rate (TPR) increases with the increase of false positive rate (FPR). When the FPR is at a low level of 0-0.2, the TPR increases slowly, ranging from 0-0.4; When FPR starts to rise from 0.2, the rising speed of TPR is accelerated, and when FPR is about 0.4, TPR reaches about 0.8; After that, FPR continued to increase, and the rising trend of TPR slowed down, finally approaching 1.0. On the whole, the chart shows typical ROC curve characteristics, which reflects the classification performance of the model under different thresholds. With the increase of the allowable false positive rate, the true positive rate also increases, but the increase amplitude decreases when the FPR is high, indicating the performance of the model in balance misjudgment and correct recognition.

**Table 10.** Countries likely to win gold for the first time.

NOC	Year	Gold	Silver	Bronze	Total
Albania	2028	0	0	2	2
Algeria*	2028	2	1	1	4
Argentina*	2028	2	1	1	4
Australasia	2028	0	0	0	0

Note: * Algeria* and Argentina* A * are the countries most likely to win the championship for the first time.

Probability map of winning gold by countries (some top countries):

Based on the logistic regression prediction model, data collection and analysis show that United States will be the first in the gold medal list of the 2028 Los Angeles Olympic Games, and all its events have considerable winning probability, with the winning probability of Athletics and Badminton even as high as 1.0. The track and field event United States has always been in a dominant position in the Olympic history, and badminton is an Olympic event that the United States has paid more attention to in recent years. Although the winning probabilities of baseball, baseball/softball, basketball, beach volleyball and boxing are lower than 0.8, they are all higher than 0.6. The winning probability of the remaining Olympic events, such as Breaking, Canoe Slalom, Canoe Sprint, Canoeing and Cycling, is between 0.6 and 0.4, and the winning probability of Dividing, Equestrianism and Fencing is between 0.2 and 0.4. It can be seen that the advantages and disadvantages of the United States coexist in Olympic events. Its advantage lies in that track and field and badminton are traditional strengths, and there are many gold medals in these two events. With excellent physical fitness and advanced training system, the United States has strong competitiveness in many of them. The United States also has outstanding strength in collective big ball games, such as basketball, volleyball and rugby, with a mature professional league system and many high-level athletes. In addition, baseball/softball and other new events in the 2028 Los Angeles Olympic Games have a broad mass base and profound cultural heritage in the United States, and American players occupy the advantage of home court. However, there are also some inferior events in the United States. In table tennis, diving and other technical events, it is difficult for the United States to compete with traditional strong teams such as China, and the results are often poor. Although the United States may win gold medals in fencing, equestrian and other events, the overall probability is small, and it does not have obvious advantages compared with some countries that focus on these events.

In the statistics of predicted medals in some countries in 2028, Algeria (Algeria) and Argentina (Argentina) are outstanding, both of which are regarded as the countries most likely to win the championship for the first time. Driven by policies and GDP, it is predicted that Algeria and Argentina will win two gold medals, one silver medal and one bronze medal in 2028, totaling four medals.

In recent years, Algeria has invested a lot of policy support in sports development. The country has made great efforts to build sports infrastructure and built a number of modern stadiums nationwide, providing athletes with a high-quality training and competition environment. At the same time, the training plan for sports talents has been implemented, and potential young talents have been selected from the youth groups for systematic professional training, and excellent athletes have been provided with opportunities for overseas exchanges and competitions. On the economic level, global macro models and analysts of Trading Economics predict that Algeria’s GDP will reach 249.02 billion US dollars by the end of 2024; In the long run, it is estimated that it will be about $256.49 billion in 2025 and $262.90 billion in 2026, which provides a strong financial guarantee for the government in sports. Argentina also has a positive policy orientation in sports development. The government attaches great importance to the inheritance and development of traditional advantageous sports such as football, and at the same time, it is constantly tapping the potential of other sports and encouraging more young people to participate in various sports. In the field of football, Argentina has a perfect youth training system, which continuously transports talents for the national team. In addition, Argentina’s GDP ranks high in South America. Although the GDP has declined in recent years, Argentina’s economy is recovering strongly, and its developed agriculture, animal husbandry and industry provide economic support for sports development.

## 4. Analysis of Great Coach Effect

It is difficult for athletes to change their nationality, but their coaches can change teams for guidance, in order to explore the role of coaches in Olympic games. Using the Synthetic Control Method model, we construct a virtual control group and two experimental groups, one is the change of the number of medals in different periods, and the other is the change of the number of medals in the same period with or without coaches, and then analyze them separately to compare the difference between the actual medal growth and the synthetic control group (the team without coaching effect):

$$\Delta Y_{\mathrm{it}}=Y_{\mathrm{it}}^{\text{treated}}-\sum_{j\in \text{control}}w_{j}Y_{\mathrm{jt}}$$ (10)

Among them, the weight is determined by minimizing the difference of pretreatment period: $W_{j}$

$$\min_{w}{∥Y_{i,\mathrm{pre}}-\sum_{j}w_{j}Y_{j,\mathrm{pre}}∥}^{2}\;s.t.\;w_{j}\geq 0,\sum w_{j}=1$$ (11)

**Figure 12.**
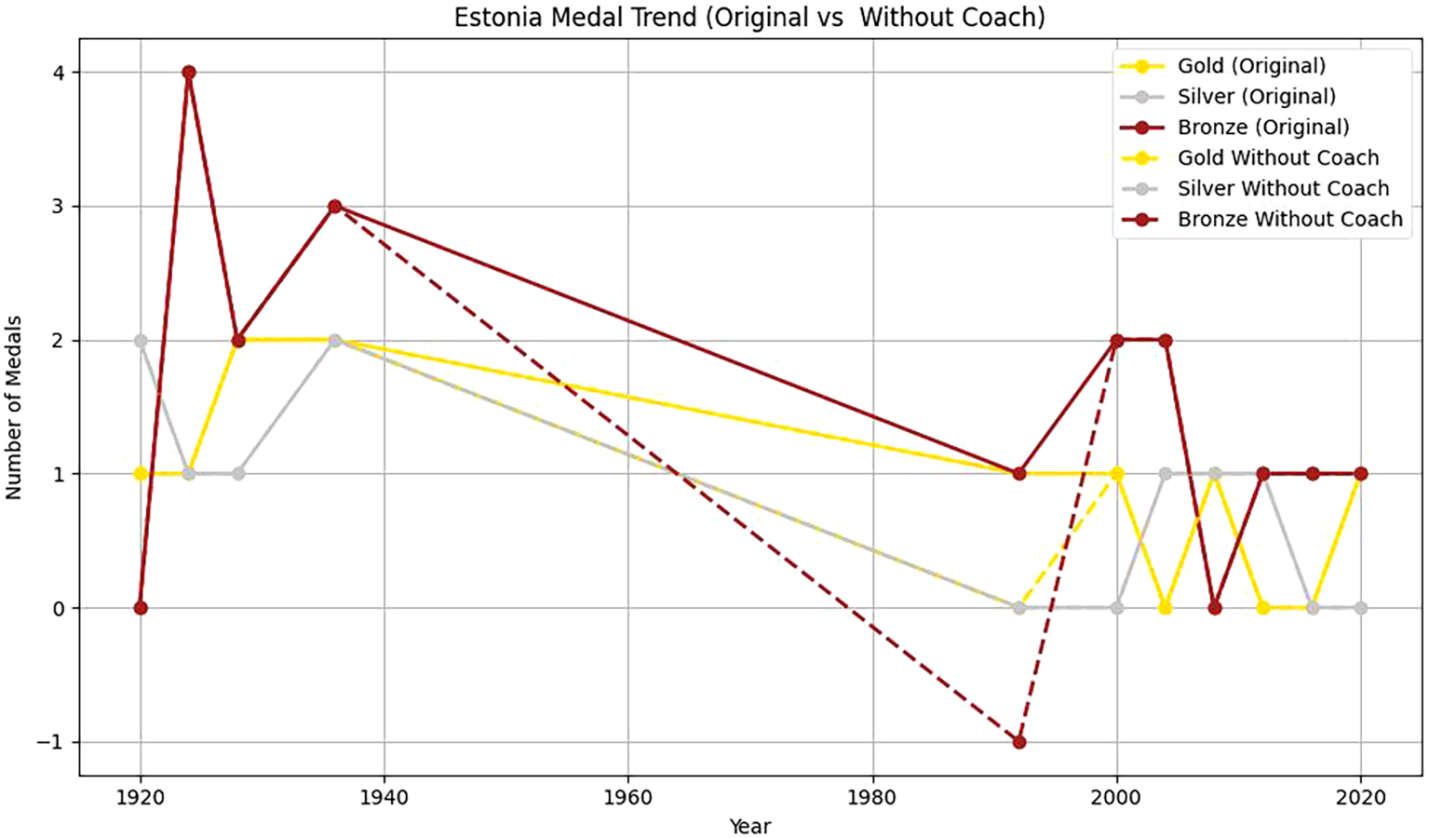
Analysis of Estonia coaching effect.

**Figure 13.**
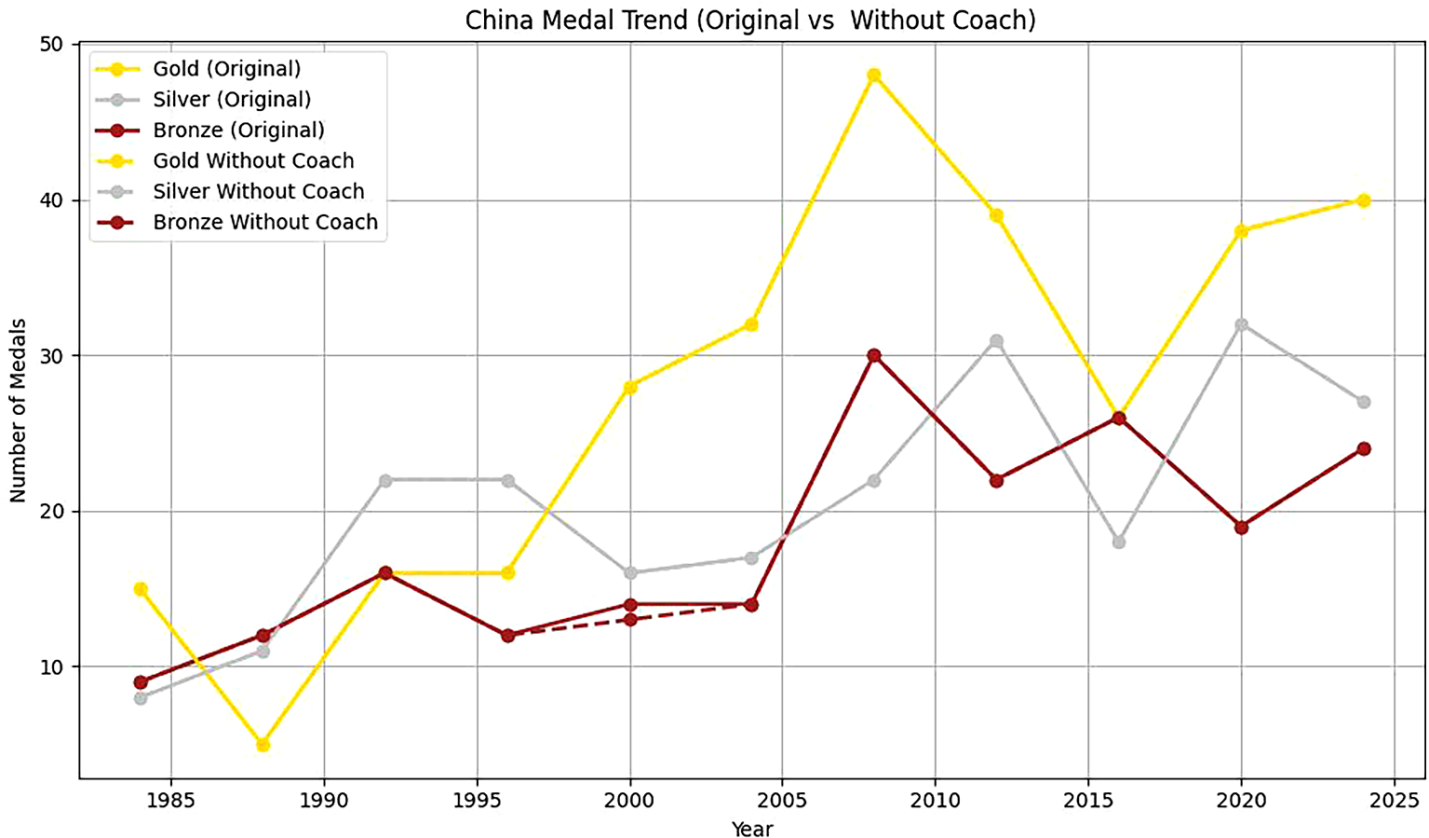
Analysis of China's coaching effect.

Two countries, Estonia and China, are selected to analyze the head coach effect. Based on Figures 12 and 13 and Table 11, it can be found that:

**Table 11.** Coach list.

Name	Team2	Year_2	Medal
Erika Salume	Estonia	1992	Gold
Qin Yiyuan	China	2000	Bronze
Tnu Tniste	Estonia	1992	Bronze
Toomas Tniste	Estonia	1992	Bronze

### 4.1 Calculation of treatment effect

The coaching effect of after intervention is defined as:

$$t>T_{0}$$ (12)

$$\Delta Y_{\mathrm{it}}=Y_{\mathrm{it}}^{\text{treated}}-Y_{\mathrm{it}}^{\text{synthetic}}$$ (13)

Namely:

$$\Delta Y_{\mathrm{it}}=Y_{\mathrm{it}}^{\text{treated}}-\sum_{j=1}^{J}\;w_{j}Y_{\mathrm{jt}}$$ (14)

Among them:

The number of medals actually observed for the treatment group $Y_{\mathrm{it}}^{\text{treated}}$

It is the predicted medal number of the synthetic control group.

$$\sum_{j=1}^{J}\;w_{j}Y_{\mathrm{jt}}$$ (15)

### 4.2 Case deduction (taking Estonia as an example)

**Data:**

Treatment group: Estonia (coach introduced in 1992)

Control group: other similar countries without coaches (such as Latvia and Lithuania)

Pretreatment period: 1980–1991

After the intervention: 1992–2020

Steps:

1. Optimizing weights: solving:
$$\min_{w}\;\sum_{t=1980}^{1991}\;{\left(Y_{\mathrm{EST},t}-\sum_{j}\;\;w_{j}Y_{j,t}\right)}^{2}$$ (16)
2. Get the optimal weight. $w_{j}^{*}$

3. Calculate the number of medals in the synthetic control group:
$$Y_{\mathrm{EST},t}^{\text{synthetic}}=\sum_{j}\;w_{j}^{*}Y_{j,t}\;\left(t\geq 1992\right)$$ (17)
4. Treatment effect:
$$\Delta Y_{\mathrm{EST},t}=Y_{\mathrm{EST},t}^{\text{actual}}-Y_{\mathrm{EST},t}^{\text{synthetic}}\;\left(t\geq 1992\right)$$ (18)

### 4.3 Results

Actual number of medals in Estonia in 1992: 1 gold+2 bronze.

The comprehensive control group predicted the number of medals: 0.5 gold+0.8 bronze.

Processing effect: medals $\Delta Y_{\mathrm{EST},1992}=\left(1-0.5\right)+\left(2-0.8\right)=1.7$ ^2^

Firstly, from 1920 to 2020, the number of gold, silver and bronze medals won by Estonia in the Olympic Games fluctuated to some extent. In 1992, Estonia won a gold medal and two bronze medals. From 2000 to 2020, Estonia’s Olympic performance was not as good as that in 1992. This shows that with a coach, Estonia has more medals overall and more balanced medal types. The coach’s guidance has a positive effect on athletes’ good performance in the competition. Without a coach, the number of medals is small, and it is mainly concentrated in some years and medal types. This further emphasizes the importance of coaches in athlete training, competition preparation and strategy formulation.

Secondly, From 1985 to 2024, the number of gold medals, silver medals and bronze medals in China showed a significant upward trend, which showed that China paid more and more attention to the international Olympic Games and invested more and more resources in its sports development. Among them, the number of gold medals and bronze medals reached the highest point in 2000, but there is still some fluctuation.

To sum up, based on the synthetic control method, combined with the virtual control group, it is found that the number of medals won by Estonia and China with head coaches is higher than that without head coaches. It shows that the great head coach effect has a significant positive effect on the performance of athletes.

## 5. Analysis of advantages and disadvantages of the model

Based on the problem background of the 2028 Los Angeles Olympic Games, this paper adopts GA-BP algorithm model and logistic regression model to analyze and predict the problems respectively. The advantages and disadvantages of the two models are as follows:

Firstly, the GA-BP algorithm model comprehensively optimizes the weights and thresholds of the BP neural network by introducing genetic algorithm, thus cleverly avoiding the inherent defect of BP neural network being prone to getting stuck in local optimal solutions. During the training phase of the model, the GA-BP algorithm model demonstrated a significantly better fit than the BP model, demonstrating significant performance advantages. Compared with the BP algorithm model, the GA-BP algorithm model achieves a significant reduction in mean square error, thereby significantly improving the prediction accuracy of the model. Secondly, the model has strong robustness. Its genetic algorithm does not depend on gradient information, and it can provide better performance and strong stability in complex or nonlinear training data when predicting the number of medals in Olympic Games. Finally, the model has strong generalization ability. The trained GA-BP algorithm model can adapt to the new data well, and its MSE reaches the minimum in the third round of training, so it has strong prediction ability for unknown data. However, this model also has some limitations. GA-BP algorithm model combines genetic algorithm and BP neural network, which leads to high computational complexity of the model. In the process of predicting Olympic medals, the calculation process of genetic algorithm is usually time-consuming, especially when dealing with large-scale data sets, which requires long training time and high computing resources. The performance of GA-BP algorithm model largely depends on the parameter settings in genetic algorithm, such as population size, crossover probability and mutation probability. The selection of these parameters has a great influence on the prediction results of the model, and improper parameter setting will affect its optimization effect, so it needs careful adjustment. Although the GA-BP algorithm model has a strong learning ability, if the training data is insufficient or there is noise, the model may be over-fitted, resulting in a decline in the ability to predict new data.

For logistic regression model, this model is a classification algorithm in machine learning, and it is widely used in practical applications. First of all, the model has strong explanatory power, and the influence degree of independent variables on dependent variables can be understood through regression coefficients. Based on predicting whether countries win gold medals for the first time in the Olympic Games and their probability, we can understand the influence of different sports on the probability of winning by regression coefficient. Secondly, the logistic regression model has high computational efficiency and is suitable for processing large-scale data. It can not only predict the data category, but also get approximate probability prediction. The prediction results can be given quickly in the probability prediction of winning gold medals in Olympic Games countries, which makes up for the shortcomings of GA-BP algorithm model. Thirdly, the logistic regression model does not require strict data distribution, and it is robust to some extent. This makes the model still maintain good prediction performance when dealing with non-normal distribution data. However, the model also has some shortcomings, which are mainly reflected in: the logistic regression model assumes that there is a linear relationship between independent variables and dependent variables, which limits the application scope of the model. In practical application, if there is a nonlinear relationship between independent variables and dependent variables, the prediction performance of logistic regression model may decline, and GA-BP algorithm model skillfully makes up for this shortcoming. At the same time, the model is sensitive to multicollinearity, and if there is a high correlation between independent variables, it may lead to instability and inaccuracy of the model. Logistic regression model is mainly used for binary classification problems, but its prediction ability for multi-classification problems or regression problems is limited. Although the model is used to predict whether countries win gold medals for the first time, it may not be accurate enough in predicting the specific number of medals.

To sum up, the combination of GA-BP algorithm model and logistic regression model adopted in this paper makes up for the shortcomings in gold medal prediction in various countries and broadens the application scope of this combination model.

## 6. Conclusion and enlightenment to that Olympic committee

Based on the in-depth analysis of the distribution and influencing factors of Olympic medals, combined with various statistical and machine learning models, this paper provides valuable insights and strategic suggestions for Olympic committees in the future. The study not only reviews the historical medal data, but also discusses the key factors that affect the medal distribution, such as the host country effect, the characteristics of sports events and the ability of the coaching team.

Firstly, this paper makes a correlation analysis, which reveals that there is a very close relationship between the number of gold medals and the total number of medals. Through the analysis of historical data, we find that the medal list not only reflects the competition of sports strength of various countries, but also reveals the resource input and strategic planning of various countries in sports development. Based on GA-BP algorithm model, this paper predicts the number of medals that countries may win in the 2028 Los Angeles Olympic Games. The results show that the United States and China, as sports powers, are expected to occupy a leading position in the gold medal list, which reflects their global influence in sports competition. Secondly, this paper also analyzes the possibility and influencing factors of countries winning gold medals for the first time in the Olympic Games through the logistic regression model. The results of the model show that there are significant differences in the winning probability of different sports, which provides an important reference for the National Olympic Committee to formulate preparation strategies. For projects with traditional advantages, the National Olympic Committee can increase investment to maintain and enhance competitiveness; For emerging or potential projects, athletes and coaches can be encouraged to actively participate in the Olympic Games through policy support and resource allocation. In addition, based on the control grouping model, this paper deeply analyzes the important influence of “great coaching effect” on athletes’ performance. Through comparative analysis, we find that countries with high-level coaches can often get better medal results in the Olympic Games. This provides an important inspiration for the National Olympic Committee in selecting and training coaches, that is, we should pay attention to the professionalism and coaching ability of the coaching team and provide more scientific and systematic training guidance for athletes.

Based on the in-depth analysis of the distribution pattern and influencing factors of Olympic medals in this study, combined with the prediction results of GA-BP algorithm model and logistic regression model, the following policy recommendations are proposed for the International Olympic Committee and sports decision-making bodies of various countries:

Firstly, optimize resource allocation and strengthen the coordinated development of advantageous projects and emerging projects. Research has shown that traditional competitive sports such as athletics and swimming in the United States, diving and table tennis in China, have made significant contributions to medals. It is recommended that countries consolidate their existing advantages through special funds, scientific research support, and the construction of high-level training bases. At the same time, it is necessary to pay attention to the dynamic potential of emerging projects such as skateboarding and rock climbing, establish flexible resource allocation mechanisms, and cultivate new medal growth points through youth selection programs, cross-border talent introduction, and policy incentives. For example, countries such as Algeria and Argentina, which are predicted to win awards for the first time, can introduce advanced training systems through international sports cooperation and focus on local characteristic projects to achieve breakthroughs.

Secondly, establish a global support network to enhance the abilities of coaches. The synthetic control model verified the positive impact of the “coach effect” on medal count, and suggested that the International Olympic Committee establish a coach cross-border exchange program to promote experience sharing and technical training, especially for developing countries. Countries should establish career development channels for coaches, improve performance evaluation systems, encourage collaboration between research institutions and coaching teams, and optimize training programs using technologies such as sports biomechanics and big data analysis. For example, Estonia’s medal breakthrough in 1992 benefited from excellent coach guidance, which can promote its “coach athlete” collaborative innovation model.

Thirdly, deepen data-driven decision-making and promote the practical application of predictive models. The GA-BP model demonstrates high accuracy and strong generalization ability in medal prediction. It is recommended that national Olympic committees establish dynamic databases that integrate multidimensional information such as historical medal data, athlete physiological indicators, and changes in competition rules, and regularly update model parameters to cope with the evolution of the competitive landscape. In addition, the logistic regression model reveals a significant impact (OR=1.011) of the “total number of participating projects” on the probability of winning, suggesting that countries need to scientifically plan their project portfolios, avoid blind expansion, and focus on high conversion areas.

Fourth, strengthen the connection between the host country effect and long-term strategy. Research has confirmed that the average number of medals won by the host country has increased by 30%. It is recommended that in the future, the host country should lay out a “home advantage” strategy in advance, including venue adaptation training, construction of audience psychological support system, and integration of local culture into event design. At the same time, it is necessary to avoid short-term benefit orientation and transform Olympic heritage into long-term sports competitiveness, such as continuously expanding talent reserves through the universalization of community sports facilities and youth sports popularization plans.

Fifth, support the fairness and sustainability of sports development in small countries. In response to the trend of small countries such as Albania and Saint Lucia winning awards for the first time, the International Olympic Committee should expand the coverage of the Olympic Solidarity Fund, provide targeted technical assistance and equipment support, and reduce competition barriers through quota adjustments and cross-border joint training. In addition, the mechanism of “North South cooperation” can be explored, with expert teams dispatched by sports powers to assist small countries in exploring advantageous projects and achieving diversified and balanced development of competitive sports.

In summary, by guiding resource allocation through scientific models, building a global coaching network, improving a data-driven decision-making system, and promoting inclusive development policies, the Olympic committees of various countries can more efficiently enhance their Olympic competitiveness and jointly promote the sustainable development of global sports.

## 7. Research shortcomings and prospects

### 7.1 Research shortcomings

1. Data Limitations

A. The study relies heavily on historical medal data and structured variables (e.g., GDP, host country status), while neglecting dynamic factors such as athlete mental states, real-time performance fluctuations, or emerging technological influences (e.g., AI-assisted training).

B. The dataset lacks granularity in capturing non-quantifiable factors, such as cultural emphasis on specific sports or geopolitical impacts on athlete participation.

2. Model Generalizability

A. The GA-BP model demonstrates strong performance in the 2028 Los Angeles Olympics prediction but lacks validation for extreme scenarios (e.g., global crises like pandemics) or long-term trends beyond a single Olympic cycle.

B. The logistic regression model assumes linear relationships between variables, limiting its applicability to complex, nonlinear interactions inherent in sports performance.

3. Parameter Sensitivity

The GA-BP model’s effectiveness depends on hyperparameter tuning (e.g., population size, mutation rate), which requires extensive computational resources and manual optimization. This limits scalability for real-time applications.

4. Ethical and Practical Constraints

A. The synthetic control method for analyzing the “great coach effect” simplifies causality by assuming homogeneity between control and experimental groups, potentially overlooking unobserved confounders (e.g., athlete genetics, institutional support).

B. Ethical considerations, such as the impact of athlete nationality changes or doping scandals, were not addressed, which may skew medal distribution patterns.

5. Scope of Analysis

The study focuses exclusively on summer Olympics, excluding winter events where medal dynamics differ significantly due to climate-specific training requirements and regional participation biases.

### 7.2 Future prospects

1. Integration of Multimodal Data

A. Incorporate real-time biometric data (e.g., wearable sensors tracking athlete physiology) and unstructured data (e.g., social media sentiment, coach feedback) to enhance prediction accuracy.

B. Develop hybrid models combining GA-BP with deep learning architectures (e.g., LSTM, transformers) to capture temporal dependencies and nonlinear relationships.

2. Model Optimization and Scalability

A. Explore metaheuristic algorithms (e.g., NSGA-II for multi-objective optimization) to automate hyperparameter tuning and reduce computational overhead.

B. Implement edge computing frameworks to enable real-time predictions during competitions, supporting tactical adjustments for teams.

3. Expanded Scope and Scenario Analysis

A. Extend the model to winter Olympics and Paralympic Games, accounting for sport-specific variables (e.g., snow conditions, adaptive equipment).

B. Simulate “black swan” scenarios (e.g., geopolitical conflicts, climate disruptions) to assess model robustness and inform contingency planning.

4. Policy-Driven Applications

A. Collaborate with international sports bodies to establish a dynamic, open-access database for global medal prediction, fostering transparency and reproducibility.

B. Develop decision-support tools for national Olympic committees, enabling scenario-based resource allocation (e.g., prioritizing high-probability events or youth development programs).

While the current study provides a robust framework for Olympic medal prediction, addressing these limitations through advanced methodologies and expanded data integration will unlock deeper insights into the interplay of sports, technology, and society. Future research should prioritize ethical AI practices and interdisciplinary collaboration to ensure models remain both accurate and equitable in an evolving global sports landscape.

## Ethics and consent

Ethical approval and consent were not required.

## Author contributions

Sanglin Zhao: Conceptualization, Data curation, formal analysis, Software, Writing–original draft, Visualization, Writing–review and editing;Jikang Cao: Conceptualization, Data curation, formal analysis, Software, Writing–original draft, Visualization, Writing–review and editing Jackon Steve: Validation, Writing review and editing. All authors have read and approved the final version of the manuscript.

## Data Availability

Figshare: Prediction of Olympic medals based on GA-BP and logistic regression model https://doi.org/10.6084/m9.figshare.28307321.v2. ^19^ The project contains the following underlying data:
•
results_of_problem_1.xlsx •
summerOly_medal_counts.csv • Processed data.xlsx • data_dictionary.csv • summerOly_programs.csv • summerOly_athletes.csv Fishare: Research on Olympic medal prediction based on GA-BP and logistic regression model Extended data. https://doi.org/10.6084/m9.figshare.28382315.v1. ^20^ This project contains the following extended data:
• Sheet 1 • Sheet 2 Figshare: STROBE checklist ‘[Research on Olympic medal prediction based on GA-BP and logistic regression model checklist’. https://doi.org/10.6084/m9.figshare.28374260.v4. ^21^
• STROBE-checklist-v4-combined (1) Data are available under the terms of the Creative Commons Zero “No rights reserved” data waiver (CC0 1.0 Public domain dedication).

## Software availability description

The software used in this study is all free and publicly available genuine software. WPS Excel (or office Excel can also replace it) is used for data processing, and WPS Excel is used for image drawing.
