# Supplementary material for: Research on Olympic medal prediction based on GA-BP and logistic regression model
Source: F1000Res. 2025 Jul 16;14:245. Originally published 2025 Feb 28. [Version 3] doi: 10.12688/f1000research.161865.3 (PMC12461187; doi:10.12688/f1000research.161865.3)
Supplement: Peer review history

# Reviewer report: Reviewer response for version 3

**DOI:** 10.5256/f1000research.184275.r414238

**Published:** 2025-09-24

**Authors:** Garrett Bullock

**Affiliations:** 1 Wake Forest School of Medicine, Winston-Salem, North Carolina, USA

Thank you for allowing my to review this paper. This study is developing prediction models on medal counts for future Olympic games. This is an interesting question. There are some questions that are required concerning the methods and construction of the paper. 

 1. The flow of the paper is not in a a traditional scientific sense. IMRD would be a much more streamlined way of putting this paper together. Right now, it is a few successive analyses with interpretation within the same sections. 

 2. There is a mixxing of prediction, association, and causal inference. You interchangeably use the words effect (e.g., coach effect, country effect) and also correlation (e.g., correlation between gold and silver and bronze medals) and prediction (e.g., the US is preicted to have the highest medal count in the next Olympics). The overall aims of this paprr is to develop and predict medal counts for future Olympics. It is advised to stick to the aims: develop the GA-BP, and forecast medal counts. Some of the data development and quality checks that are in the earlier methods are good to keep, but the focus should be on development and forecasting of medal counts. 

 3. Deep learning requires massive amounts of data. The data itself is not the largest. This can be OK, particularly in a proof of concept perspective. However, within the prediction metrics, the variance of these predictions needs to be included, not just the point estimates. This will allow for improved interpretation. 

 4. This study should be informed of reporting by TRIPOD AI+.

Is the work clearly and accurately presented and does it cite the current literature?

Partly

If applicable, is the statistical analysis and its interpretation appropriate?

Partly

Are all the source data underlying the results available to ensure full reproducibility?

Yes

Is the study design appropriate and is the work technically sound?

Partly

Are the conclusions drawn adequately supported by the results?

Partly

Are sufficient details of methods and analysis provided to allow replication by others?

Partly

Reviewer Expertise:

Prediction, Epidemiology, AI, Sport, Sport Medicine

I confirm that I have read this submission and believe that I have an appropriate level of expertise to confirm that it is of an acceptable scientific standard, however I have significant reservations, as outlined above.

# Reviewer report: Reviewer response for version 3

**DOI:** 10.5256/f1000research.184275.r398763

**Published:** 2025-07-25

**Authors:** C.V. Suresh Babu

**Affiliations:** 1 Hindustan Institute of Technology and Science, Chennai, Tamil Nadu, India

All suggestions have been **satisfactorily incorporated and justified** in the revised manuscript. The authors not only addressed the feedback but provided extended and contextual elaborations, particularly on the synthetic control method and the rationale for authorship changes.

Is the work clearly and accurately presented and does it cite the current literature?

Partly

If applicable, is the statistical analysis and its interpretation appropriate?

Yes

Are all the source data underlying the results available to ensure full reproducibility?

Partly

Is the study design appropriate and is the work technically sound?

Yes

Are the conclusions drawn adequately supported by the results?

Partly

Are sufficient details of methods and analysis provided to allow replication by others?

Partly

Reviewer Expertise:

AI, ML

I confirm that I have read this submission and believe that I have an appropriate level of expertise to confirm that it is of an acceptable scientific standard.

# Reviewer report: Reviewer response for version 2

**DOI:** 10.5256/f1000research.179311.r385994

**Published:** 2025-06-25

**Authors:** C.V. Suresh Babu

**Affiliations:** 1 Hindustan Institute of Technology and Science, Chennai, Tamil Nadu, India

Responses to Review Questions

1. **Is the work clearly and accurately presented and does it cite the current literature?**
**Partly**
  The study is presented with reasonable clarity, but several sections, particularly the introduction and methodology, would benefit from better articulation of objectives and more detailed citations of recent work. Some key claims (e.g., the coach effect) lack robust sourcing.
2. **Is the study design appropriate and is the work technically sound?**
**Yes**
  The dual-model approach combining GA-BP and logistic regression is appropriate for the multifaceted nature of Olympic medal prediction. The use of the synthetic control model adds depth to the coach effect analysis.
3. **Are sufficient details of methods and analysis provided to allow replication by others?**
**Partly**
  While the core methodology is outlined, important implementation details are omitted:
  - Network architecture specifics (e.g., number of layers/neurons, activation functions)
  - Genetic algorithm parameters (e.g., population size, mutation rate)
  - Training-validation split, cross-validation methods
    These gaps hinder full reproducibility.
4. **If applicable, is the statistical analysis and its interpretation appropriate?**
**Yes**
  The use of R², MSE, MAE, and Evar for model evaluation is standard and appropriate. Logistic regression outputs are well interpreted, and ROC/AUC analysis is correctly applied.
5. **Are all the source data underlying the results available to ensure full reproducibility?**
**Partly**
  The manuscript claims to provide datasets on Figshare, but in-text descriptions of these datasets (structure, variables) and analysis code are missing. Full transparency would require direct links and documentation on how each dataset was used.
6. **Are the conclusions drawn adequately supported by the results?**
**Partly**
  The conclusions align with the presented results. However, the causal claims regarding the coach effect should be toned down. The synthetic control method assumes group similarity, but the paper does not verify covariate balance or control for potential confounders.

Is the work clearly and accurately presented and does it cite the current literature?

Partly

If applicable, is the statistical analysis and its interpretation appropriate?

Yes

Are all the source data underlying the results available to ensure full reproducibility?

Partly

Is the study design appropriate and is the work technically sound?

Yes

Are the conclusions drawn adequately supported by the results?

Partly

Are sufficient details of methods and analysis provided to allow replication by others?

Partly

Reviewer Expertise:

AI, ML

I confirm that I have read this submission and believe that I have an appropriate level of expertise to confirm that it is of an acceptable scientific standard, however I have significant reservations, as outlined above.

# Response

**Published:** 2025-07-05

**Authors:** Jackon Steve

**Affiliations:** School of Management, University of Khartoum, Khartoum, Khartoum, Sudan

We appreciate your feedback on the revisions. Overall, the newly submitted manuscript focuses on strengthening the literature review and discussion of synthetic control methods and head coach effects in the introduction section. We have added code to the data involved in the article.

# Reviewer report: Reviewer response for version 1

**DOI:** 10.5256/f1000research.177956.r369223

**Published:** 2025-03-07

**Authors:** Yiquan Wang, Tin-Yeh Huang

**Affiliations:** 1 Xinjiang University, Xinjiang, China; 2 Shenzhen X-Institute, Shenzhen, Guangdong, China; 3 Department of Industrial and System Engineering, The Hong Kong Polytechnic University, Hong Kong SAR, China

The manuscript presents an interesting study that combines a genetic algorithm–optimized backpropagation neural network (GA-BP) and logistic regression to predict Olympic medal outcomes and evaluate the impact of coaching. While the multi-model approach is innovative, several revisions are recommended for clarity, reproducibility, and balanced interpretation.

 Focus and Literature: The introduction should clearly state the research objectives and hypotheses. It would be beneficial to explain how GA-BP improves upon traditional methods and why logistic regression is used alongside the neural network. Additionally, updating the literature review to include recent advances in sports analytics and machine learning would better contextualize the study.

 Methodological Detail: Key aspects such as data preprocessing steps, neural network architecture (e.g., layers, neurons, activation functions, learning rate), and genetic algorithm settings (e.g., population size, mutation rates) need more thorough explanation. Providing detailed model validation processes (like cross-validation and sensitivity analyses) would also help ensure reproducibility.

 Data and Code Transparency: The manuscript should include a clear description of available datasets (e.g., file structures and variables) and ensure that analysis scripts and code are accessible via a public repository. This transparency is essential for replicability and further research.

 Interpretation and Conclusions: While the results are promising, the causal claims regarding the “great coach effect” should be presented more cautiously. Separating correlation from causation and tempering policy recommendations based on the current methodological limitations would strengthen the discussion.

 Overall, addressing these points would enhance the manuscript's clarity, methodological rigor, and scientific contribution.

Is the work clearly and accurately presented and does it cite the current literature?

Partly

If applicable, is the statistical analysis and its interpretation appropriate?

Yes

Are all the source data underlying the results available to ensure full reproducibility?

Partly

Is the study design appropriate and is the work technically sound?

Yes

Are the conclusions drawn adequately supported by the results?

Partly

Are sufficient details of methods and analysis provided to allow replication by others?

Partly

Reviewer Expertise:

artificial intelligence，machine Learning, deep Learning

We confirm that we have read this submission and believe that we have an appropriate level of expertise to confirm that it is of an acceptable scientific standard, however we have significant reservations, as outlined above.
